# The Validity and Reliability of the My Jump Lab App for the Measurement of Vertical Jump Performance Using Artificial Intelligence

**DOI:** 10.3390/s24247897

**Published:** 2024-12-10

**Authors:** Carlos Balsalobre-Fernández, Daniel Varela-Olalla

**Affiliations:** Applied Biomechanics and Sport Technology Research Group, Autonomous University of Madrid, 28049 Madrid, Spain; daniel.varela@uam.es

**Keywords:** computer vision, physical performance, smartphone, markerless motion tracking, vertical jump

## Abstract

The countermovement jump (CMJ) is a widely used test to assess lower body neuromuscular performance. This study aims to analyze the validity and reliability of an iOS application using artificial intelligence to measure CMJ height, force, velocity, and power in unloaded and loaded conditions. Twelve physically active participants performed 12 CMJs with external loads ranging from 0% to 70% of their body mass while being simultaneously monitored with a pair of force platforms and the My Jump Lab application. The scores for jump height, mean propulsive force, velocity, and power between devices were compared for validity and reliability purposes. The force platform and the application showed a high association (r > 0.91, *p* < 0.05) for measuring CMJ height, force, velocity, and power. Small and no statistically significant differences (*p* < 0.05) were observed in most loading conditions. Both instruments showed high reliability (Cronbach’s α > 0.93, Coefficient of variation < 6%) for measuring the different trials performed by each participant. The My Jump Lab application was shown to be valid and reliable for measuring CMJ height, force, velocity, and power in both loaded and unloaded jumps, eliminating the problems associated with the cost and portability of force plates for daily practice.

## 1. Introduction

The assessment of lower-limb neuromuscular capacities is one of the most common evaluations in athletic and non-athletic populations, due to its important role in sports performance and overall health [1,2,3,4]. In particular, vertical jump tests are widely used for this purpose due to their ability to demonstrate force, velocity, and power production capabilities in a simple, time-efficient, and nearly non-invasive way [5,6,7]. For example, 9 out of 10 professional football teams use them as a regular test in their performance monitoring structure [8]. Over the past 15 years, several investigations have explored the ability of vertical jump tests to estimate the mechanical parameters underpinning the features of power production, showing that concentric force, velocity, and power production can be assessed in a valid, reliable, and simple way without the need to use laboratory-grade equipment [4,9,10,11]. Samozino et al. [12] showed that simple equations using vertical jump height and basic anthropometric data can estimate lower-limb force, velocity, and power production with a high degree of precision in comparison with a force platform. Thanks to this, lower-limb force–velocity profiling, i.e., the determination of maximal force, velocity, and power production capabilities in jumping, has rapidly become a common assessment in field settings, especially in clubs, clinics, or institutions without the resources to obtain force platforms. In this sense, different low-cost alternatives have been tested for the measurement of vertical jump height over the last decade [13,14,15,16,17,18].

Several investigations have demonstrated the validity and reliability of smartphone applications to estimate vertical jump height using slow-motion video analysis [13,14,18,19]. Specifically, the My Jump app (then renamed to My Jump 2, and more recently, to My Jump Lab) calculates jump height by measuring flight time, and has been validated in a wide range of populations, from elite athletes to the elderly, and using different smartphone models and camera configurations [20,21,22,23,24,25]. Thanks to advances in smartphone technologies, a newer version of the My Jump Lab app for iOS was developed, including computer vision and machine learning algorithms designed for the real-time measurement of countermovement jump (CMJ) height. Recent investigations have observed that this artificial intelligence method is valid and reliable for the measurement of CMJ height in comparison with a force platform [26,27,28]. However, no studies have analyzed the ability of any smartphone app to estimate vertical jump force, velocity, and power production using Samozino’s equations [12]. Given that Samozino’s equations derive such variables from jump height and anthropometric measurements, we hypothesize that the jump height estimation provided by My Jump Lab can yield valid and reliable computations of force, velocity, and power during a CMJ. Additionally, to the best of our knowledge, no studies have explored the validity and reliability of the My Jump Lab application for measuring vertical jump performance during loaded jumps.

Thus, the purpose of the present investigation is to explore the validity and reliability of the My Jump Lab app for iOS for the measurement of CMJ height, force, velocity, and power production in loaded and unloaded jumps.

## 2. Materials and Methods

### 2.1. Experimental Approach to the Problem

We aimed to compare CMJ height measured by My Jump Lab and force platforms (which was considered the gold standard for our purposes) in a range of external loads. Additionally, we wanted to compare the CMJ mean propulsive force, velocity, and power computed from Samozino’s method [12] by using the app’s or the force plate’s derived jump height as the input. This biomechanical model enables the estimation of force, velocity, and power by inputting jump height and basic anthropometric data, such as body mass, into its validated equations. To do this, four sets of three CMJs using loads ranging from 0 to 70% of participants’ body mass were simultaneously recorded by dual force platforms (Hawkin Dynamics, Westbrook, ME, USA) and the My Jump Lab (Madrid, Spain) app installed on an iPhone 14 Pro running iOS 18.1 (Apple Inc., Cupertino, CA, USA) during a single testing session. A total of 144 repetitions were registered, and data between both devices were compared for validity and reliability purposes.

### 2.2. Subjects

Twelve healthy male sport science students with no history of injuries in the past 12 months were recruited (mean ± SD: age = 22.1 ± 2.5 years; mass = 74.2 ± 7.4 kg). Subjects were instructed to refrain from strenuous exercise for two days prior to the testing session. All individuals were attired in a t-shirt, shorts, and running shoes, lacking any specific guidelines regarding the clothes’ fit to their body or color. Participants were informed of the potential risks and benefits of the study prior to data collection.

### 2.3. Procedures and Instruments

Each participant completed a standardized 10 min warm-up consisting of 5 min of dynamic stretching, forward and lateral lunges, and bodyweight squats. Participants then performed three practice trials of the CMJ at their perceived maximal effort. Finally, they performed three maximal CMJs on a Smith machine using the empty barbell as an external load. For data collection, all jumps were performed on dual force platforms (Hawkin Dynamics, Westbrook, ME, USA) whilst being concurrently recorded by My Jump Lab installed on an iPhone 14 Pro (Apple Inc., Cupertino, CA, USA). All data collected from the force plates (FP) at 1000 Hz were transmitted via a Bluetooth connection to an Android tablet using Hawkin Dynamics proprietary software v. 9.5.0. The app used the iPhone camera and artificial intelligence algorithms to detect the entire body of the participants and perform markerless motion tracking at a rate of 60 Hz at a resolution of 1080 p, which was then used for the calculations of CMJ performance as described and validated elsewhere [28]. To register CMJ height from the app, the iPhone was mounted to a camera tripod to record the frontal plane of the participant, at a height such that the focal center of the video screen passed approximately 1.2 m above the floor with the participant centered horizontally on the screen while remaining visible during the entire flight phase of the CMJ. Participants were asked to place their hands on their hips, to look at the camera, and to remain steady until the recording started. The force platform calculated jump height from take-off velocity using the impulse–momentum theorem.

All participants stepped onto dual force platforms and were required to perform four sets of three maximal-effort CMJs with 90 s rest intervals between jumps, and 5 min rests between sets. The first set of each participant consisted of 3 unloaded CMJs, while the three remaining sets consisted of jump squats performed on a Smith machine using different loads corresponding to 20, 50, and 70% of their body mass in an approximately random order. Upon stepping onto the force platforms, participants were required to stand motionless for 2 s so that their body weight could be accurately determined. Their legs were required to remain fully extended during the flight phase of the jump. Finally, mean propulsive force, velocity, and power were computed with Samozino’s equations using jump height from My Jump Lab and the force platforms.

### 2.4. Statistical Analyses

All values were initially recorded as means ± standard deviations (SD) in Microsoft Excel. The normality of the data was confirmed using a Shapiro–Wilk test (*p* > 0.05). To determine the concurrent validity between measurement methods, Pearson’s correlation coefficients (r) were calculated. Within-session reliability was computed for both measurement methods using the coefficient of variation (CV), calculated as follows: (SD/average) * 100 and Cronbach’s alpha. CV values less than 10% were deemed acceptable [5]. Systematic bias between methods was determined by a paired samples *t*-test, with statistical significance set at *p* < 0.05. The practical significance between the FP and My Jump Lab app was also determined using Cohen’s *d* effect sizes with the 95% confidence interval (CI). These were interpreted in line with suggestions by Rhea [29] relative to the “recreationally trained” sample in the present study: <0.35 = trivial; 0.35–0.79 = small; 0.80–1.49 = moderate; and >1.50 = large. The limits of agreement corresponding to 1.96 SD of the bias (LoA) and 95% CI between the FP and the My Jump Lab app were determined from Bland–Altman plots. The statistical software JASP 0.17.1 for macOS was used.

## 3. Results

### 3.1. Concurrent Validity and Bias Determination

When analyzing the concurrent validity of My Jump Lab for the measurement of CMJ height and mean propulsive force, velocity, and power in comparison with the force platform-derived metrics, almost perfect correlations were observed (r = 0.971–0.984, *p* < 0.001). The paired samples *t*-test and Cohen’s *d* effect size revealed small-to-moderate or no statistically significant differences (*p* < 0.05) between the force platform and My Jump Lab for the measurement of CMJ height and mean propulsive force, power, and velocity in most loading conditions. See Table 1 for more information.

### 3.2. Reliability

Similar levels of reliability were observed between My Jump Lab and the force platform for the measurement of the three CMJs of each participant during each set, as revealed by the high Cronbach’s alpha scores (α > 0.9) and low coefficients of determination (CV < 10%). See Table 2 for more information.

### 3.3. Bland–Altman Analysis

**Figure 1:**
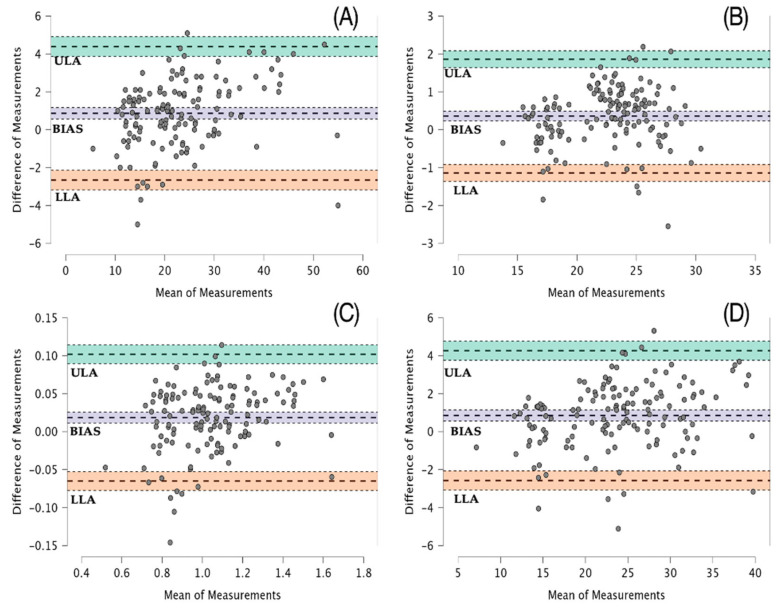
The Bland–Altman plots with their limits of agreement (LoA) for the measurement of CMJ height (**A**) and mean propulsive force (**B**), velocity (**C**), and power (**D**) between the force plate and My Jump Lab. The dashed line in the middle of each plot represents the Bland–Altman bias, while the upper and lower dashed lines represent the upper and lower LoA (ULA and LLA, respectively). The colored areas represent the 95%CI of the bias and upper and lower LoA.

Figure 1A–D shows the Bland–Altman plots with the distribution of the difference between the force platform and My Jump Lab across the range of each variable. The bias between instruments was shown to be homoscedastic (i.e., not proportionally increasing or decreasing as the variable changes), as revealed by the coefficient of determination of the regression line of the Bland–Altman plots for the measurement of CMJ height (R^2^ = 0.09), mean propulsive force (R^2^ = 0.01), velocity (R^2^ = 0.05), and power (R^2^ = 0.07). See Table 3 for more details about the bias and limits of agreement of each Bland–Altman plot.

## 4. Discussion

We aimed to analyze the validity and reliability of a previously validated smartphone app powered by artificial intelligence (AI) algorithms for the measurement of vertical jump performance using a range of external loads, including unloaded CMJs and increasingly loaded CMJs executed on a Smith machine. Specifically, the app analyzed in the present investigation included a recently developed feature that estimates jump height by performing markerless motion tracking thanks to computer vision, a set of artificial intelligence techniques designed to recognize patterns in images or videos [28,30,31,32]. To the best of our knowledge, three different investigations have analyzed the validity and reliability of this AI feature of My Jump Lab recently. We first conducted a proof-of-concept investigation where we developed and tested an update to the previously validated My Jump Lab app [28], which used computer vision to measure CMJ height in real time. Then, two independent groups confirmed the validity and reliability (*r* > 0.9, *ICC* > 0.9) of this AI feature in different populations, including elite athletes [26,27]. However, no studies have analyzed how the app performs when measuring countermovement jumps with external loads. While other jump height measurement systems do not seem affected by external loads (since they measure flight time or take-off velocity) [6,14,33], computer vision-based systems like the one used in this investigation might be. My Jump Lab uses Apple’s Vision Framework, which includes proprietary machine learning models trained to detect human bodies [28]. Computer vision algorithms must recognize a human body in the live feed of the iPhone camera at 60 Hz. This means the presence of other humans or interfering elements can pose a challenge to the app’s performance, especially since artificial intelligence techniques based on computer vision rely on discriminating different features from multiple pixels in each image [30]. The computational power and diversity of body types can also influence the accuracy of computer vision models in detecting human poses in real time. Consequently, future studies with larger and more diverse sample sizes, including individuals from various age groups, genders, and ethnicities, are necessary. Furthermore, considering that the application can be installed on any device running iOS 15 or newer, it implies that even an 8-year-old iPhone can utilize the artificial intelligence feature tested in the present investigation. Therefore, additional studies are required to compare less capable devices with newer versions to ascertain whether a difference exists in the association between My Jump Lab and a force platform. Despite these challenges, our results demonstrate a high and similar correlation between My Jump Lab and the force platform in measuring CMJ performance under both unloading and loading conditions, indicating that the presence of the Smith machine and bar on the participant’s shoulders did not affect the algorithm’s ability to detect and track their movements during jumps.

To the best of our knowledge, this investigation presents the first attempt to validate and assess the reliability of the application for estimating mean propulsive force, velocity, and power, derived from jump height using Samozino’s simple equations. Sixteen years ago, Samozino et al. [12] validated a simple method for estimating vertical jump force, velocity, and power. This method has gained popularity as an alternative to measuring force–velocity profiles in field conditions because it can estimate force, velocity, and power production capabilities by only measuring jump height and basic anthropometric measurements. Our results demonstrate that the computed mean propulsive force, velocity, and power using Samozino’s equations, obtained by entering push-off distance, body mass, and jump height from force platforms or the My Jump Lab app, are highly correlated (*r* > 0.91). Furthermore, when analyzing the reliability of the app for measuring the three trials in each loading condition with each participant, very high levels of reliability (Cronbach’s α > 0.93, CV < 6%) were observed for both instruments. These results align with previous investigations that have shown similar levels of validity and reliability for My Jump Lab in measuring CMJ height compared to a force platform [20,34,35,36].

When comparing the force platform and My Jump Lab for measuring the unloaded CMJ height, our study found a mean difference of 2.0 ± 1.3 cm. The app consistently provided higher scores than the force platform (Bland–Altman coefficient of determination *R*^2^ < 0.1). These results align with those of Tan et al. [27], who also noted that My Jump Lab’s AI feature tends to overestimate CMJ height by 2 cm, systematically. Several studies have examined the use of slow-motion video recording to measure jump height compared to reference methods over the past decade, with My Jump 2, the previous version of the current My Jump Lab app, being the most tested smartphone app [13,14,17,18]. Slow-motion methods involve recording a jump video and manually selecting take-off and landing frames to calculate flight time. Various devices, operating systems, and camera configurations have been tested, showing that 240 Hz slow-motion analysis (the standard in most smartphones) yields lower absolute differences compared to the AI feature investigated here [18,21,22]. However, the reliability scores appear similar to those in our study (Cronbach’s α > 0.9). For instance, Rogers et al. [37] observed a mean bias of 0.59 ± 0.32 cm between My Jump 2 and a force platform for measuring CMJ height in trained athletes. The primary limitation of slow-motion video analysis is its inability to provide real-time feedback since the measurement must be performed after recording the video. In contrast, the AI method offers a time-efficient alternative for practitioners seeking real-time feedback for their athletes. Researchers, on the other hand, who prioritize the highest level of accuracy and do not require live data may prefer the slow-motion approach. However, no studies have directly compared the accuracy, validity, and reliability of the slow-motion and AI measurements in My Jump Lab with the gold standard. Future research comparing both methods to a force platform could highlight the differences between them and help practitioners select the most appropriate option for their specific context.

The findings of our study collectively demonstrate that the My Jump Lab application for iOS serves as a valid and reliable alternative for measuring countermovement jump height and computing the mean propulsive force, velocity, and power using Samozino’s equations. These results may be of interest to practitioners seeking to monitor CMJ height and lower-limb force, velocity, and power capabilities in a simple and cost-effective manner, since other tools commonly used for this purpose, like force plates, have the main limitations of being excessively expensive and impractical for efficient daily use when time is restricted, or large groups should be measured at once.

## Figures and Tables

**Table 1:** The mean values ± SD of the CMJ metrics using the force platform and My Jump Lab, the effect size of the difference between instruments (Cohen’s *d*), and Pearson’s product–moment correlation coefficient (*r*) at each tested load.

Variable	Force Platform	My Jump Lab	*r* (95% CI)	Mean Difference (±SD)	*d* (95% CI)
Height (cm)					
Pooled	22.5 ± 9.2	23.4 ± 9.8	0.984 (0.977–0.988)	1.6 ± 1.1	0.483 (0.307–0.657)
Unloaded jump	33.3 ± 10.2	34.7 ± 10.6	0.982 (0.963–0.991)	2.0 ± 1.3	0.675 (0.286–1.055) *
Load 1	21.6 ± 7.1	22.2 ± 8.0	0.974 (0.950–0.987)	1.6 ± 1.2	0.340 (0.002–0.674)
Load 2	19.2 ± 5.4	20.2 ± 5.7	0.971 (0.943–0.985)	1.4 ± 0.9	0.700 (0.331–1.061) *
Load 3	17.0 ± 3.8	17.6 ± 4.2	0.910 (0.830–0.954)	1.4 ± 1.2	0.301 (−0.035–0.633)
Mean propulsive force (N/kg)					
Pooled	22.3 ± 3.7	22.7 ± 3.8	0.979 (0.971–0.985)	0.68 ± 0.49	0.471 (0.296–0.645)
Unloaded jump	19.64 ± 4.70	20.08 ± 5.00	0.993 (0.987–0.997)	0.67 ± 0.45	0.690 (0.321–1.050) *
Load 1	21.73 ± 3.47	22.01 ± 3.71	0.980 (0.960–0.990)	0.68 ± 0.44	0.360 (0.020–0.694)
Load 2	22.70 ± 3.57	23.15 ± 3.62	0.984 (0.968–0.992)	0.68 ± 0.45	0.690 (0.322–1.051) *
Load 3	23.73 ± 3.84	23.96 ± 3.81	0.970 (0.941–0.985)	0.73 ± 0.62	0.253 (−0.081–0.583)
Mean propulsive velocity (m/s)					
Pooled	1.03 ± 0.2	1.05 ± 0.22	0.981 (0.973–0.986)	0.03 ± 0.02	0.432 (0.258–0.605)
Unloaded jump	1.12 ± 0.44	1.14 ± 0.20	0.997 (0.994–0.999)	0.03 ± 0.02	0.644 (0.281–1.000) *
Load 1	1.01 ± 0.17	1.02 ± 0.20	0.972 (0.946–0.986)	0.04 ± 0.03	0.241 (−0.093–0.570)
Load 2	0.96 ± 0.13	0.98 ± 0.14	0.969 (0.939–0.984)	0.04 ± 0.02	0.660 (0.295–1.017) *
Load 3	0.90 ± 0.10	0.92 ± 0.11	0.913 (0.836–0.955)	0.03 ± 0.02	0.288 (−0.047–0.620)
Mean propulsive power (W/kg)					
Pooled	23.3 ± 6.8	24.1 ± 7.3	0.971 (0.960–0.979)	1.56 ± 1.14	0.485 (0.309–0.659)
Unloaded jump	23.91 ± 11.2	25.01 ± 11.95	0.991 (0.983–0.996)	1.72 ± 1.19	0.663 (0.298–1.021) *
Load 1	22.55 ± 6.84	23.21 ± 7.53	0.975 (0.952–0.987)	1.56 ± 1.02	0.380 (0.039–0.715)
Load 2	22.22 ± 6.12	23.21 ± 6.29	0.974 (0.950–0.987)	1.42 ± 0.96	0.699 (0.330–1.060) *
Load 3	21.86 ± 5.52	22.40 ± 5.62	0.933 (0.872–0.966)	1.60 ± 1.35	0.264 (−0.070–0.595)

* Statistically significant difference using paired-samples *t*-test (*p* < 0.05).

**Table 2:** The coefficient of variation (mean ± SD) and Cronbach’s α for the measurement of the three different trials of each participant during each set.

Variable	CV (%)	Cronbach’s α
Force platform		
Height (cm)	5.0 ± 4.1	0.985
Mean propulsive force (N/kg)	2.2 ± 1.9	0.944
Mean propulsive velocity (m/s)	2.9 ± 3.4	0.983
Mean propulsive power (W/kg)	4.5 ± 3.6	0.985
My Jump Lab		
Height (cm)	5.8 ± 4.6	0.981
Mean propulsive force (N/kg)	2.5 ± 2.2	0.935
Mean propulsive velocity (m/s)	3.4 ± 4.2	0.977
Mean propulsive power (W/kg)	5.3 ± 4.0	0.980

**Table 3:** Bland–Altman bias and limits of agreement (LoA) with 95% confidence interval for the measurement of CMJ height, and mean propulsive force, power, and velocity.

Bias and LoA	Point Value	Lower 95% CI	Upper 95% CI
Height (cm)			
Mean difference + 1.96 SD	4.393	3.873	4.914
Mean difference	0.868	0.567	1.168
Mean difference −1.96 SD	−2.657	−3.178	−2.137
Mean propulsive force (N/kg)			
Mean difference + 1.96 SD	1.862	1.640	2.084
Mean difference	0.361	0.233	0.489
Mean difference − 1.96 SD	−1.140	−1.362	−0.918
Mean propulsive velocity (m/s)			
Mean difference + 1.96 SD	0.102	0.090	0.114
Mean difference	0.018	0.011	0.026
Mean difference − 1.96 SD	−0.065	−0.077	−0.053
Mean propulsive power (W/kg)			
Mean difference + 1.96 SD	4.267	3.762	4.772
Mean difference	0.846	0.555	1.138
Mean difference − 1.96 SD	−2.574	−3.079	−2.069

## Data Availability

The raw data are available with the following Document Object Identifier (DOI): https://doi.org/10.6084/m9.figshare.27888879.

## References

[1] Fry A.C. (2004). The Role of Resistance Exercise Intensity on Muscle Fibre Adaptations. Sports Med..

[2] Folland J.P., Williams A.G. (2007). The Adaptations to Strength Training. Sports Med..

[3] Stamatakis E., Lee I.-M., Bennie J., Freeston J., Hamer M., O’Donovan G., Ding D., Bauman A., Mavros Y. (2018). Does Strength Promoting Exercise Confer Unique Health Benefits? A Pooled Analysis of Eleven Population Cohorts with All-Cause, Cancer, and Cardiovascular Mortality Endpoints. Am. J. Epidemiol..

[4] Morin J.B., Samozino P. (2016). Interpreting Power-Force-Velocity Profiles for Individualized and Specific Training. Int. J. Sports Physiol. Perform..

[5] Cormack S.J., Newton R.U., McGuigan M.R. (2008). Neuromuscular and Endocrine Responses of Elite Players to an Australian Rules Football Match. Int. J. Sports Physiol. Perform..

[6] Bishop C., Turner A., Jordan M., Harry J., Loturco I., Lake J., Comfort P. (2022). A Framework to Guide Practitioners for Selecting Metrics during the Countermovement and Drop Jump Tests. Strength Cond. J..

[7] Kozinc Ž., Žitnik J., Smajla D., Šarabon N. (2022). The Difference between Squat Jump and Countermovement Jump in 770 Male and Female Participants from Different Sports. Eur. J. Sport Sci..

[8] Asimakidis N.D., Bishop C.J., Beato M., Mukandi I.N., Kelly A.L., Weldon A., Turner A.N. (2024). A Survey into the Current Fitness Testing Practices of Elite Male Soccer Practitioners: From Assessment to Communicating Results. Front. Physiol..

[9] Samozino P., Rejc E., Di Prampero P.E., Belli A., Morin J.B. (2012). Optimal Force-Velocity Profile in Ballistic Movements—Altius: Citius or Fortius?. Med. Sci. Sports Exerc..

[10] Nibali M.L., Chapman D.W., Robergs R.A., Drinkwater E.J. (2013). A Rationale for Assessing the Lower-Body Power Profile in Team Sport Athletes. J. Strength Cond. Res..

[11] Agar-Newman D.J., Tsai M.-C., Klimstra M. (2022). The Validity of Applying a Simple Three-Factor Computational Model to Calculate Force, Power, and Speed Using Hexagonal Bar Jumps. J. Strength Cond. Res..

[12] Samozino P., Morin J.-B., Hintzy F., Belli A. (2008). A Simple Method for Measuring Force, Velocity and Power Output during Squat Jump. J. Biomech..

[13] Montalvo S., Gonzalez M.P., Dietze-Hermosa M.S., Eggleston J.D., Dorgo S. (2021). Common Vertical Jump and Reactive Strength Index Measuring Devices: A Validity and Reliability Analysis. J. Strength Cond. Res..

[14] Balsalobre-Fernández C., Glaister M., Lockey R.A. (2015). The Validity and Reliability of an IPhone App for Measuring Vertical Jump Performance. J. Sports Sci..

[15] Martínez-Martí F., González-Montesinos J., Morales D., Santos J., Castro-Piñero J., Carvajal M., Palma A. (2016). Validation of Instrumented Insoles for Measuring Height in Vertical Jump. Int. J. Sports Med..

[16] Pueo B., Lopez J.J., Jimenez-Olmedo J.M. (2019). Audio-Based System for Automatic Measurement of Jump Height in Sports Science. Sensors.

[17] Balsalobre-Fernández C., Tejero-González C.M., del Campo-Vecino J., Bavaresco N. (2014). The Concurrent Validity and Reliability of a Low-Cost, High-Speed Camera-Based Method for Measuring the Flight Time of Vertical Jumps. J. Strength Cond. Res..

[18] Sharp A.P., Cronin J.B., Neville J. (2019). Using Smartphones for Jump Diagnostics: A Brief Review of the Validity and Reliability of the My Jump App. Strength Cond. J..

[19] Bishop C., Jarvis P., Turner A., Balsalobre-Fernandez C. (2022). Validity and Reliability of Strategy Metrics to Assess Countermovement Jump Performance Using the Newly Developed My Jump Lab Smartphone Application. J. Hum. Kinet..

[20] Stojiljković N., Stanković D., Pelemiš V., Čokorilo N., Olanescu M., Peris M., Suciu A., Plesa A. (2024). Validity and Reliability of the My Jump 2 App for Detecting Interlimb Asymmetry in Young Female Basketball Players. Front. Sports Act. Living.

[21] Wirtz S., Julian R., Schmale R., Eils E. (2024). Concurrent Validity and Reliability of In-Field Vertical Jump Performance Measures on Sand Surfaces. J. Strength Cond. Res..

[22] Pueo B., Hopkins W., Penichet-Tomas A., Jimenez-Olmedo J. (2023). Accuracy of Flight Time and Countermovement-Jump Height Estimated from Videos at Different Frame Rates with MyJump. Biol. Sport.

[23] Bogataj Š., Pajek M., Hadžić V., Andrašić S., Padulo J., Trajković N. (2020). Validity, Reliability, and Usefulness of My Jump 2 App for Measuring Vertical Jump in Primary School Children. Int. J. Environ. Res. Public Health.

[24] Coswig V., Silva A.D.A.C.E., Barbalho M., De Faria F.R., Nogueira C.D., Borges M., Buratti J.R., Vieira I.B., Román F.J.L., Gorla J.I. (2019). Assessing the Validity of the MyJump2 App for Measuring Different Jumps in Professional Cerebral Palsy Football Players: An Experimental Study. JMIR mHealth uHealth.

[25] Cruvinel-Cabral R.M., Oliveira-Silva I., Medeiros A.R., Claudino J.G., Jiménez-Reyes P., Boullosa D.A. (2018). The Validity and Reliability of the “ My Jump App” for Measuring Jump Height of the Elderly. PeerJ.

[26] Şentürk D., Yüksel O., Akyildiz Z. (2024). The Concurrent Validity and Reliability of the My Jump Lab Smartphone App for the Real-Time Measurement of Vertical Jump Performance. Proc. Inst. Mech. Eng. Part P J. Sports Eng. Technol..

[27] Tan E.C.H., Weng Onn S., Montalvo S. (2024). Measuring Vertical Jump Height With Artificial Intelligence Through a Cell Phone: A Validity and Reliability Report. J. Strength Cond. Res..

[28] Balsalobre-Fernández C. (2024). Real Time Estimation of Vertical Jump Height with a Markerless Motion Capture Smartphone App: A Proof-of-Concept Case Study. Proc. Inst. Mech. Eng. Part P J. Sports Eng. Technol..

[29] Rhea M.R. (2004). Determining the Magnitude of Treatment Effects in Strength Training Research Through the Use of the Effect Size. J. Strength Cond. Res..

[30] Naik B.T., Hashmi M.F., Bokde N.D. (2022). A Comprehensive Review of Computer Vision in Sports: Open Issues, Future Trends and Research Directions. Appl. Sci..

[31] Khanal S.R., Paulino D., Sampaio J., Barroso J., Reis A., Filipe V. (2022). A Review on Computer Vision Technology for Physical Exercise Monitoring. Algorithms.

[32] Barris S., Button C. (2008). A Review of Vision-Based Motion Analysis in Sport. Sports Med..

[33] Glatthorn J.F., Gouge S., Nussbaumer S., Stauffacher S., Impellizzeri F.M., Maffiuletti N.A. (2011). Validity and Reliability of Optojump Photoelectric Cells for Estimating Vertical Jump Height. J. Strength Cond. Res..

[34] Haynes T., Bishop C., Antrobus M., Brazier J. (2019). The Validity and Reliability of the My Jump 2 App for Measuring the Reactive Strength Index and Drop Jump Performance. J. Sports Med. Phys. Fitness.

[35] Barbalho M., Kleiner A.F.R., Callegari B., de Lima R.C., da Silva Souza G., de Athayde Costa e Silva A., Coswig V.S. (2021). Assessing Interlimb Jump Asymmetry in Young Soccer Players: The My Jump 2 App. Int. J. Sports Physiol. Perform..

[36] Whiteley I., Sideris V., Kotsifaki R., King E., Whiteley R. (2023). The MyJump App Is a Valid Method of Assessing and Classifying Limb Symmetry During Recovery from Anterior Cruciate Ligament Reconstruction. Int. J. Sports Phys. Ther..

[37] Rogers S.A., Hassmén P., Hunter A., Alcock A., Crewe S.T., Strauts J.A., Gilleard W.L., Weissensteiner J.R. (2019). The Validity and Reliability of the MyJump2 Application to Assess Vertical Jumps in Trained Junior Athletes. Meas. Phys. Educ. Exerc. Sci..

